# Obesity and risk of pancreatic cancer among postmenopausal women: the Women's Health Initiative (United States)

**DOI:** 10.1038/sj.bjc.6604487

**Published:** 2008-07-15

**Authors:** J Luo, K L Margolis, H-O Adami, A LaCroix, W Ye

**Affiliations:** 1 Institute of Social Development and Public Policy, Beijing Normal University, Beijing, China; 2 Department of Medical Epidemiology and Biostatistics, Karolinska Institutet, Stockholm, Sweden; 3 HealthPartners Research Foundation, Minneapolis, MN, USA; 4 Department of Epidemiology, Harvard School of Public Health, Boston, MA, USA; 5 Department of Epidemiology, University of Washington, Seattle, WA, USA

**Keywords:** pancreatic cancer, obesity, central obesity, body mass index, waist-to-hip ratio

## Abstract

A total of 138 503 women in the Women's Health Initiative in the United States were followed (for an average of 7.7 years) through 12 September 2005 to examine obesity, especially central obesity in relation to pancreatic cancer (*n*=251). Women in the highest quintile of waist-to-hip ratio had 70% (95% confidence interval 10–160%) excess risk of pancreatic cancer compared with women in the lowest quintile.

Pancreatic cancer ranks as the fourth leading cause of cancer death in the United States (Ekbom and Hunter, 2007). Besides tobacco smoking and chronic pancreatitis (Lowenfels *et al*, 1999), little is known of its aetiology, but recently, increasing evidence has suggested that obesity is a risk factor (Berrington de Gonzalez *et al*, 2003; Larsson *et al*, 2007). However, in most studies, the association seems to be weaker in women – who tend to gain weight more peripherally – than in men, suggesting that central adiposity may be a stronger risk factor for pancreatic cancer than body mass index (BMI). Few studies have investigated this aspect and the findings are inconsistent (Larsson *et al*, 2005; Sinner *et al*, 2005; Ansary-Moghaddam *et al*, 2006; Berrington de Gonzalez *et al*, 2006).

We have used the large prospective Women's Health Initiative (WHI) study, with measured anthropometric factors, including hip and waist circumference, to examine the relationship between BMI, central adiposity, and pancreatic cancer risk.

## Materials and methods

### Women's Health Initiative

The WHI, an ongoing, ethnically and geographically diverse, multi-centre clinical trial (CT) and observational study (OS), was designed to address some of the major causes of morbidity and mortality in postmenopausal women. Briefly, a total of 161 808 women aged 50–79 years were recruited at 40 clinical centres throughout the United States from 1 September 1993 through 1998. The WHI includes three overlapping CT components (hormone trial, dietary modification trial, and calcium/vitamin D supplementation trial) and an OS. All participants in WHI gave informed consent and were followed prospectively. Details of the scientific rationale, eligibility requirements and baseline characteristics of the participants in the WHI have been published elsewhere (Hays *et al*, 2003; Jackson *et al*, 2003; Langer *et al*, 2003; Ritenbaugh *et al*, 2003; Stefanick *et al*, 2003).

The following participants were excluded from the original cohort of 161 808 : 14 849 with a history of cancer (except non-melanoma skin cancer) at baseline, 668 with no follow-up, 7491 with missing values of main exposures and confounders (including weight, height, waist circumference, hip circumference, smoking, and diabetes), and 297 (0.18%) with waist-to-hip ratio (WHR) values of beyond 4 s.d. (WHR<0.4 or WHR>1.2), leaving 138 503 women for analysis.

All exposures in our analyses were collected at baseline for all participants. During the baseline clinical visit, trained and certified staff performed anthropometric measurements, including height, weight, hip and waist circumferences, and blood pressure. Body mass index was calculated as weight in kilograms divided by the square of height in metres. Waist circumference at the natural waist or narrowest part of the torso and hip circumference at the maximal circumference were measured to the nearest 0.1 cm. WHR was computed as the ratio of these two measurements. Weight changes during the participant's adult lives were obtained by self-report questionnaire, categorized as weight stayed stable (within 10 lb), steady gain in weight, lost weight as an adult and kept it off, and weight has gone up and down again by more than 10 lb.

Information on demographic characteristics, medical history, and personal habits (lifestyle) were obtained by interview or by self-report using standardized questionnaires including age at enrolment (<55, 55–59, 60–64, 65–69, 70–74, 75–), smoking status (never, past, current) with information on how many years since quitting for past smokers and how many cigarettes smoked per day for ever smokers, physical activity and history of hypertension and diabetes while not pregnant.

Women in the CT were followed through regularly scheduled examinations to ensure timely ascertainment of updated medical histories. All women in the CT were expected to attend annual clinical visits, with intermediate 6-month mail, phone or clinical contacts. The OS participants were contacted annually by mailed self-administered questionnaires. The completion rate of OS annual questionnaires was 93–96%. In this analysis, all participants were followed up until 12 September 2005. Initial reports of cancer were ascertained by self-administered questionnaires, and all self-reports of pancreatic cancer were confirmed by review of medical records, including pathology reports (if a biopsy or resection was done).

### Statistical analyses

The hazard ratio (HR) for pancreatic cancer was estimated using the Cox proportional hazards model. Different study cohorts (participation in OS or CTs, and different treatment assignments for all three CTs) were treated as strata in the model. In the multivariable models, we adjusted for age, smoking status (never, former smoking (quitted time⩾30 years, 20–29, 10–19, and <10 years), and current smoking (<4, 5–14, 15–24, 25 and more cigarettes per day)). As diabetes could be in the causal pathway between obesity and risk, we performed analyses (both adjusted and unadjusted) for this factor.

We treated anthropometric measures as categorical (in quintiles) variables in the regression models, except BMI which was categorized (<22, 22–<25, 25–<30, 30–<35, and ⩾35 kg m^−2^). Tests for trend were performed by creating a continuous variable from the medians of the categories. In addition, to eliminate undiagnosed cases that might have experienced weight loss before completing the baseline questionnaires, we also performed analyses that excluded the first 2 years of follow-up. The proportional assumption was satisfied for all exposure variables of interest and potential confounding variables based on graphs of scaled Schoenfeld residuals (Hess, 1995).

## Results

As of 12 September 2005 with an average 7.7 years of follow-up, 251 incident cases of pancreatic cancer had been identified. Characteristics at baseline of selected variables by quintile of WHR are shown in Table 1. Compared with women with lower WHR, those with higher WHR were older, non-white, and less educated. Women with higher WHR were also more likely to be past or current smokers and tended to smoke more cigarettes per day and to have quit more recently, to have higher total energy intake, be less physically active, and have higher prevalence of diabetes and hypertension. WHR was positively associated with weight, BMI, waist and hip circumference.

Among the tested anthropometric variables, only WHR was significantly associated with the risk of pancreatic cancer. After adjusting for potential confounders, women in the highest quintile of WHR had 70% (95% CI 10–160%) excess risk compared with women in the lowest quintile of WHR. When WHR was analyzed as a continuous variable, risk increased by 27% (95% CI 7–50%) per 0.1 increase. No association was observed between pancreatic cancer risk and other anthropometric variables, including height, BMI, hip circumference, waist circumference, and weight changes during adult life (Table 2).

Finally, we repeated all the above analyses with the exclusion of the first 2 years of follow-up; findings remained broadly similar to those from the full analyses with RR=1.6 (95% CI: 1.0–2.6) comparing women in the highest to the lowest of WHR.

## Discussion

In this large prospective study, we observed that central obesity measured by high WHR, rather than general obesity measured by high BMI, was associated with an increased risk of developing pancreatic cancer among postmenopausal women.

Our result is consistent with most earlier studies for central adiposity and pancreatic cancer (Larsson *et al*, 2005; Patel *et al*, 2005; Ansary-Moghaddam *et al*, 2006; Berrington de Gonzalez *et al*, 2006), although not all (Sinner *et al*, 2005). In particular, the large prospective European prospective investigation into cancer and nutrition (EPIC) study (Berrington de Gonzalez *et al*, 2006) also observed significant increased risk associated with higher WHR (HR=1.24 (1.04–1.48) per 0.1 increase of WHR), but not with BMI. In fact, there was a weak or no association among women in all but 4 (Michaud *et al*, 2001; Calle *et al*, 2003; Pan *et al*, 2004; Patel *et al*, 2005) of the 16 studies published since 2000 on the association between the risk of pancreatic cancer and BMI that included women (Coughlin *et al*, 2000; Gapstur *et al*, 2000; Nilsen and Vatten, 2000; Hanley *et al*, 2001; Michaud *et al*, 2001; Wolk *et al*, 2001; Calle *et al*, 2003; Pan *et al*, 2004; Eberle *et al*, 2005; Fryzek *et al*, 2005; Larsson *et al*, 2005; Patel *et al*, 2005; Rapp *et al*, 2005; Sinner *et al*, 2005; Lin *et al*, 2007; Nothlings *et al*, 2007).

The link between obesity and pancreatic cancer – similar to the plausible mechanism between diabetes and pancreatic cancer – may arise as a result of elevated fasting and postprandial glucose concentration, hyperinsulinemia, or both (Everhart and Wright, 1995; Gapstur *et al*, 2000; Batty *et al*, 2004; Jee *et al*, 2005). Many experimental studies (Pour and Stepan, 1984; Pour *et al*, 1990; Schneider *et al*, 2001; Wang *et al*, 2003; Hennig *et al*, 2004) and observational studies (Everhart and Wright, 1995; Gapstur *et al*, 2000; Huxley *et al*, 2005; Stolzenberg-Solomon *et al*, 2005) support the biological plausibility of higher insulin concentrations and insulin resistance in promoting pancreatic cancer development. If the induced insulin resistance is the underlying mechanism through which obesity increases the risk, then it is not surprising that we observed a stronger association with WHR abdominal adiposity than with BMI among postmenopausal women, because central adiposity is more strongly associated with glucose intolerance and increased insulin levels (Carey *et al*, 1997; Van Pelt *et al*, 2001; Sierra-Johnson *et al*, 2004; Tanko *et al*, 2004). In addition, the body fat distribution changes significantly following menopause, with a shift from preferential storage in gluteal/femoral regions to abdominal depots. Thus, our finding further suggests that the central adiposity is a better predictor of disease risk than BMI in postmenopausal women (Van Pelt *et al*, 2001).

Strengths of our study include the prospective design, the large size of the cohort, the reasonably large number of cases, the high prevalence of obesity, including central adiposity, the detailed information on potential confounders, and the precise measurement of anthropometric factors. Measurement rather than self-reporting is particularly important for waist and hip circumferences, which are likely to be reported less accurately than height and weight. There is a possibility of misclassification among the cases, given the difficulty in diagnosis of this disease. However, this misclassification is likely to be non-differential with respect to anthropometric measurements, which may make our results conservative. A second possibility was weight loss because of undiagnosed disease, but results did not change after excluding the first 2 years of follow-up. It is unlikely that pancreatic cancer advanced enough to cause weight loss would remain undiagnosed for more than 2 years.

In conclusion, our large prospective study shows that increased central adiposity was associated with an increased risk of developing pancreatic cancer among postmenopausal women.

## Figures and Tables

**Table 1:** Baseline characteristics of participants in relation to WHR (quintiles) among 138 503 postmenopausal women^a^

	**WHR (quintile, range)**				
**Variable**	**1 (0.40–0.75)**	**2 (0.75–0.79)**	**3 (0.79–0.82)**	**4 (0.82–0.87)**	**5 (0.87–1.20)**
Total number of women	27 707	27 694	27 736	27 651	27 715
Age at baseline (mean, years)	61.4	62.7	63.3	63.7	64.2
White, non-Hispanic-ethnicity (%)	86.9	84.3	82.0	79.6	79.6
College graduate or above education (%)	48.1	43.1	38.3	34.9	31.3
					
*Ever smoking* (%)					
Past	37.6	39.2	40.1	41.1	42.8
Quit <20 years(%)	41.4	44.3	46.5	51.3	54.5
					
Current	5.0	6.7	7.2	7.8	8.9
⩾15 cigarettes day^−1^ (%)	40.4	43.3	48.5	49.3	53.9
					
*Dietary intake (mean)*					
Total energy intake (kcal day^−1^)	1580	1611	1638	1679	1727
Fruit and vegetable(median servings per day)	4.3	4.2	4.1	3.9	3.9
Physical activity (METs per week)	15.2	13.8	12.3	11.0	9.7
Diabetes mellitus (%)	1.4	2.3	4.0	6.9	14.2
Hypertension (%)	19.4	26.0	32.5	39.5	48.8
					
Weight (kg, mean)	65.5	69.1	72.8	77.1	81.8
Height (cm, Mean)	162.4	162.0	161.7	161.5	161.5
BMI (kg m^−2^, mean)	24.9	26.3	27.8	29.5	31.3
Waist circumference (mean, cm)	73.8	80.2	85.6	91.8	100.8
Hip circumference (mean, cm)	103.3	104.5	106.5	108.7	109.5
WHR (mean)	0.71	0.77	0.80	0.85	0.92
					
*Type of weight change*					
Steady gain in weight	23.6	30.3	34.4	38.0	39.6
Lost weight and kept it off	4.3	3.0	2.2	1.8	1.4
Weight upon and down (more than 10 lb)	27.6	31.0	34.3	37.4	41.1

BMI=body mass index; MET=metabolic equivalent tasks; WHR=waist-to-hip ratio.

a All comparisons of cohort characteristics by waist-to-hip levels are significantly different, with *P*<0.0001 based on *χ*^2^ test for categorical variables and analysis of variance (ANOVA) test for continuous variables.

**Table 2:** Age-adjusted and multivariate-adjusted HR of pancreatic cancer by baseline measures of adiposity among 138 503 postmenopausal women

**Variable**	**No. of cases**	**Age-adjusted HR (95% CI)**	**Multi-adjusted HR (95% CI)^a^**	**Multi-adjusted HR (95% CI)^b^**
*Height (cm)*				
1 (100.0–156.4, 153.6)	63	Reference	Reference	Reference
2 (156.5–160.1, 158.5)	54	1.0 (0.7–1.4)	0.9 (0.6–1.3)	0.9 (0.6–1.3)
3 (160.2–163.4, 161.9)	48	0.9 (0.6–1.3)	0.8 (0.6–1.2)	0.8 (0.6–1.2)
4 (163.5–167.0, 165.1)	43	0.9 (0.6–1.2)	0.8 (0.5–1.1)	0.8 (0.5–1.1)
5 (167.1–212.0, 170.0)	43	0.9 (0.6–1.3)	0.9 (0.6–1.3)	0.9 (0.6–1.3)
*P* (trend)		0.4	0.3	0.3
				
*BMI (kg m*^−*2*^)				
<22.0	25	0.8 (0.5–1.2)	0.8 (0.5–1.2)	0.8 (0.5–1.2)
22.0–24.9	62	Reference	Reference	Reference
25.0–29.9	84	0.9 (0.6–1.2)	0.9 (0.6–1.2)	0.9 (0.6–1.2)
30.0–34.9	56	1.2 (0.8–1.7)	1.1 (0.8–1.6)	1.1 (0.7–1.5)
35.0–	24	0.9 (0.6–1.4)	0.9 (0.5–1.4)	0.8 (0.5–1.3)
*P* (trend)		0.4	0.5	0.9
				
*Hip (cm) quintile (range, median)*				
1 (40.0–96.9, 93.0)	52	Reference	Reference	Reference
2 (97.0–101.9, 99.0)	58	1.2 (0.8–1.7)	1.2 (0.8–1.8)	1.2 (0.8–1.8)
3 (102.0–107.1, 104.3)	50	1.0 (0.6–1.4)	1.0 (0.6–1.4)	0.9 (0.6–1.4)
4 (107.1–115.0, 111.0)	42	0.9 (0.6–1.3)	0.9 (0.6–1.3)	0.8 (0.6–1.3)
5 (115.1–200.0, 122.5)	49	1.1 (0.7–1.6)	1.1 (0.7–1.6)	1.0 (0.7–1.5)
*P* (trend)		0.7	0.6	0.4
				
*Waist (cm) quintile (range, median)*				
1 (35.0–74.5, 70.5)	41	Reference	Reference	Reference
2 (74.6–81.0, 78.0)	50	1.2 (0.8–1.7)	1.1 (0.7–1.7)	1.1 (0.7–1.7)
3 (81.1–88.0, 85.0)	46	1.1 (0.7–1.6)	1.0 (0.7–1.6)	1.0 (0.7–1.6)
4 (88.1–97.4, 92.4)	63	1.5 (1.0–2.2)	1.4 (1.0–2.1)	1.4 (0.9–2.0)
5 (97.5–194.2, 105.0)	51	1.3 (0.9–1.9)	1.2 (0.8–1.8)	1.1 (0.7–1.6)
*P* (trend)		0.1	0.2	0.6
Waist as continuous (per 10 cm)	251	1.10 (1.00–1.20)	1.08 (0.98–1.18)	1.05 (0.95–1.15)
				
*WHR quintile (range, median)*				
1 (0.40–0.75, 0.72)	34	Reference	Reference	Reference
2 (0.75–0.79, 0.77)	47	1.3 (0.8–2.0)	1.3 (0.8–1.9)	1.2 (0.8–1.9)
3 (0.79–0.82, 0.80)	44	1.2 (0.7–1.8)	1.1 (0.7–1.7)	1.1 (0.7–1.7)
4 (0.82–0.87, 0.84)	48	1.2 (0.8–1.9)	1.2 (0.7–1.8)	1.1 (0.7–1.7)
5 (0.87–1.20, 0.91)	78	2.0 (1.4–3.0)	1.8 (1.2–2.8)	1.7 (1.1–2.6)
*P* (trend)		0.0003	0.002	0.01
WHR as continuous (per 0.1)	251	1.38 (1.17–1.63)	1.32 (1.12–1.56)	1.27 (1.07–1.50)
				
*Type of weight change*				
Stable weight	85	Reference	Reference	Reference
Steady gain in weight	77	0.9 (0.7–1.2)	0.9 (0.7–1.2)	0.9 (0.6–1.2)
Lost weight and kept it off	5	0.7 (0.3–1.6)	0.6 (0.3–1.6)	0.6 (0.3–1.5)
Weight up and down (more than 10 lb)	83	1.0 (0.7–1.3)	0.9 (0.7–1.3)	0.9 (0.7–1.2)

CI=confidence interval; HR=hazard ratio; WHR=waist-to-hip ratio.

a Adjusted variables included age, different treatment assignments in clinical trials, and smoking status (never, former smoking (quitted time⩾30years, 20-<30, 10-<20, <10 years), and current smoking (<4, 5–14, 15–24, 25 and more cigarettes per day)).

b Further adjusted for diabetes history at baseline.
